# Research on influencing factors of college teachers’ second child fertility intentions——Taking Jinan as an example

**DOI:** 10.1371/journal.pone.0299838

**Published:** 2024-05-21

**Authors:** Yanling Yao

**Affiliations:** School of Information Engineering, Shandong Management University, Jinan, Shandong, China; University of Salamanca, SPAIN

## Abstract

**Background:**

Fertility intentions, as a direct driver of fertility behavior, play an important role in the implementation of national fertility policy and population development. This study explored the influencing factors of college teachers’ second child fertility intentions in Jinan, China on the basis of TPB.

**Methodology/Principal findings:**

Based on the theory of planned behavior, this paper employs basic characteristics analysis, difference analysis, and factor analysis related to the fertility intentions of the participants. Analysis found participants between 31 and 40 years old had the highest second child fertility intentions, and participants with a college-age first child had the lowest second child fertility intentions. Attitude and subjective norms had a positive impact on second child fertility intentions, and policy awareness had a positive impact on attitude, which indirectly affected second child fertility intentions. Subjective norms had the greatest influence on second child fertility intentions, followed by attitude, and policy awareness had the least influence on second child fertility intentions.

**Significance/Future research:**

The findings of this paper can enrich the theoretical research on fertility intentions, and also provide more optimal practical references for the formulation and propagation of China’s fertility policy as well as the improvement of the division of family roles in China. Future research can further explore the impact of fertility policy on the fertility intentions of other groups.

## Introduction

A country’s population characteristics are directly related to its social development and the well-being of its people; population also has strategic significance [1–5]. Devising fertility policies appropriate to a country’s social development has become a topic of much sociology and demography research [6,7]. As a direct driver of fertility behavior, fertility intentions are a key consideration for fertility policy [8,9]. Fertility intentions are people’s subjective intentions or attitudes toward childbearing, and they have an important predictive effect on fertility behavior [10–12]; they determine, to a certain extent, the fertility level and population development of a country or region [11]. Scholars in different countries and regions are increasingly studying fertility intentions [13–21]. Therefore, fertility intentions are of great practical significance for a country or region. Research related to fertility intentions is of even greater significance for low-fertility countries—the number of which continues to rise as fertility rates in many countries around the world decline [10].

In recent years, a rapidly aging population, low birth rate, and shrinking labor force in China have created a series of issues for its social development. To address these issues, China has begun to implement a series of policy measures. China implemented a two-child policy in 2016, which allows a couple to have two children, with the main purpose of addressing the issues of an aging population and a declining birth rate. The two-child policy has been the subject of a good deal of research [22–26]. Although scholars have not reached a consensus on the specific role of the two-child policy [27], some judge the two-child policy to be a success and argue that by maintaining the current level of fertility intentions, the two-child policy is conducive to alleviating China’s aging problem [28], solving the problem of the sex ratio imbalance [26], and significantly reducing the cesarean section rate [29]. Other measures that should also be taken to maximize the impact of the two-child policy include comprehensive reforms at the supply level [30] and policies to promote the growth of human capital [31]. Some scholars also posit that the effect of the two-child policy will be apparent only after a significant delay [28], so further research on the second child fertility intentions is still needed.

The previous studies analyzing various factors affecting fertility intentions provide a solid theoretical and practical foundation for this study. Scholars have conducted many studies on the factors influencing the fertility intentions of the general population to have a second child [32,33]. To date, there have been few studies on the fertility intentions of specific groups of people, and only a small number of scholars have studied the fertility intentions of specific samples of people such as early childhood teachers [34], college students [35], and transient population [36]. The study of special groups in this paper is motivated by concerns about policy implications, improvements in policy design. The issues related to the second child fertility intentions of specific groups of people need to be further studied.

Therefore, this paper takes college teachers in China as the research object. College teachers are more likely to have a more positive attitude towards children’s education, invest more in education, and exhibit greater understanding and acceptance of fertility policies and regulations than the general population. Based on the theory of planned behavior (hereinafter referred to as TPB), this paper investigates the factors that affect the second child fertility intentions of college teachers in China, and proposes related measures. This study is the first to analyze the factors influencing Chinese college teachers’ second child fertility intentions based on data from a sample of college teachers in Jinan, China and uses a research methodology based on TPB and considers policy awareness, parental help, cost factor, and utility factor to form a model of factors influencing college teachers’ second child fertility intentions.

This research can enrich the body of knowledge related to fertility intentions and also provide theoretical references for future research on the fertility intentions of other groups. The findings of this paper also offer insight for the formulation and implementation of China’s fertility policy, and for the improvement of the division of family roles in China.

## Literature review

### Theory of planned behavior

TPB was first proposed by Ajzen on the basis of the theory of reasoned action. According to this theory, intention is mainly affected by attitudes, subjective norms and perceived behavior control [37]. Attitude refers to an internal evaluation of an individual that performing the behavior or attaining the goal will have positive or negative outcomes for them. Subjective norms refer to an individual’s perception of the psychological support or pressure exerted by their close members to actually adopt a certain behavior. Perceived behavior control refers to the perceived difficulty or ease of a person’s behavior in achieving a goal [38]. This theory has been widely applied in a wide range of disciplines. For example, TPB is used to study energy-saving behavior [39–41], product purchase behavior [42], learning management system adoption behavior [43,44], tourism behavior [45], the sustainable use behavior of shared bicycle users [46], consumers’ purchase behavior of green products [47], accommodation choice behavior [48], and career choice behavior [49]. Some scholars have also used TPB to study the factors influencing fertility intentions [50,51].

### Hypotheses development

The factors affecting fertility intentions are very complex and have been the subject of a great deal of scholarly research [34,52,53]. Research indicates that at the micro level, fertility intentions are related to factors such as working conditions [54], economic conditions [34,52,55], housing conditions [53], gender role attitudes [56,57], and family support [58], while at the macro level, fertility intentions are related to family policy and fertility policy, among other factors. Some scholars argue that economic conditions have a substitutionary effect on fertility intentions, i.e., the better a couple’s economic conditions, the lower their fertility intentions [59]. Other research indicates that the higher the employment uncertainty, the lower the fertility intentions [60,61]. This effect is consistent across highly educated people and young people with uncertain employment [62,63]; therefore, some scholars have proposed that the perception of unemployment elasticity has a strong predictive effect on fertility intentions [64]. However, other scholars have proffered the opposite view, arguing that an uncertain work environment can lead to higher fertility [65]. Regarding housing conditions on fertility intentions, some scholars posit that non-urban families and rental families have higher fertility intentions [66], and some scholars note that migrant workers who own their own housing are less willing to have a second child [67].

Higher identification with traditional gender roles such as in China and Egypt is associated with higher fertility intentions [55,68]; however, some research has reached the opposite conclusion—that more equal gender role attitudes lead to higher fertility intentions [69,70]. Some research suggests that gender role attitudes have an opposite effect on the fertility intentions for a first child and a second child, and gender role attitudes have a positive effect on the fertility intentions for a first child, while they have a negative effect on the fertility intentions for a second child [56]. Some scholars have proposed that the greater the difference in gender role attitudes between couples, the lower the fertility intentions [71]. With regard to the impact of family policy on fertility intentions, some scholars argue that the more perfect family policy, the higher the affected population’s fertility intentions [72]; the same conclusion has also been reached among the women under 35 years old [73] and the men between 31 and 36 years old [74] who have a second child. As for research on the impact of social and family support on fertility intentions, scholars have reached a consensus that the higher the level of social and family support, the higher the fertility intentions [75–78]. As for the impact of gender-based division of labor on fertility intentions, some scholars argue that a division of labor allocating a heavier load to women will reduce fertility intentions [79], and some scholars argue that a restrictive condition should be added to this conclusion, that is, the gender of the child should not be considered [80]. Some research indicates that the higher the consistency of intention and values between couples, the higher the fertility intentions [81]; however, other research did not find a link between higher consistency between couples and higher fertility intentions [82].

Some research has examined the impact of fertility policy on fertility intentions. Some scholars argue that fertility policy is a decisive factor in affecting fertility intentions [83], and the better the fertility policy, the more positively it affects fertility intentions, which is more obvious among young people [84]. As for research on the impact of social security on fertility intentions, research generally indicates that the more effective the social security system, the lower the fertility intentions, so social security seems to have a substitution effect on fertility intentions [59]. In addition to the above factors, the frequency of media and internet use [55,68], pet attachment [85], the intimacy of spousal relationships [86], childhood family background [87], family systems [58], union formation [88], and climate-related and environmental factors [89–91] can have an impact on fertility intentions.

Some research has focused on fertility intentions among specific populations. Studies have examined the fertility intentions of HIV-infected populations in Africa [92–96] and migrant populations in China [97]. Some scholars have studied the fertility intentions of college students [98,99] and disabled women [100].

## Conceptual model

According to TPB, attitude, subjective norms and perceived behavior control together affect individual behavior intention. Scholars have identified or derived numerous influencing factors based on TPB. The specific influencing factors need to be comprehensively considered according to different research fields and research objects. In essence, fertility behavior is a social activity, which can be affected by various subjective and objective factors [11]. Table 1 lists the influencing factors and their reference adopted by previous fertility intentions research. This paper takes college teachers in Jinan, China as the research object and studies their second child fertility intentions by referring to the influencing factors in Table 1. In addition to attitude, subjective norms, and perceived behavior control, other influencing factors are policy awareness [84], parental help [52], cost factor [101], and utility factor [102].

**Table 1:** Influencing factors and reference sources in the study of fertility intentions.

Dimension	Influencing factor
Basic demographic characteristics	Gender [54,103,104]
	Age [38,105–107]
	Health status [38,105,108]
	Education level [38,105–109]
	Housing conditions [38,105,108]
	Gender of the first child [110]
	Age of the first child [111]
Influencing factors	Attitude [38,105–109,112]
	Subjective norms [38,105–109,112]
	Perceived behavior control [38,105–107,109,112]
	Policy awareness [58,84]
	Parental help [52,113]
	Cost factor [101,102]
	Utility factor [102]

Based on Table 1, influencing factors suitable for this study were selected and a model of factors influencing college teachers’ second child fertility intentions was constructed. The model included seven basic demographic characteristics, shown in Table 1.

For influencing factors, TPB mainly includes three factors: attitude, subjective norms and perceived behavior control. Therefore, these three influencing factors are included first. Some studies have found that policy awareness and acceptance are the first step for the promulgation and implementation of public policies [84]. Other studies have noted that help from parents or grandparents can reduce the cost of childcare in terms of time and energy [113]. Scholars note there are cost and utility factors in fertility intentions [102]. Therefore, this paper also incorporates four influencing factors: policy awareness, parental help, cost factor, and utility factor, for a total of eight influencing factors for this study, seven of which are independent variables (attitude, subjective norms, perceived behavior control, policy awareness, parental help, cost factor, and utility factor), with fertility intentions as the dependent variable.

The seven independent variables may have direct or indirect effects on the dependent variable, and there may also be interactions among the independent variables. The seven basic demographic characteristics may also have moderating effects. Therefore, this paper proposes the following 12 hypotheses:

Hypothesis 1 (H1): Policy awareness has a positive impact on attitude. Among the demographic characteristics, age, gender, and education level have moderating effects.
Hypothesis 2 (H2): Policy awareness has a positive impact on fertility intentions. Among the demographic characteristics, age, gender, and education level have moderating effects.
Hypothesis 3 (H3): Cost factor has a negative impact on attitude. Among the demographic characteristics, housing conditions have a moderating effect.
Hypothesis 4 (H4): Cost factor has a negative impact on perceived behavior control. Among the demographic characteristics, housing conditions have a moderating effect.
Hypothesis 5 (H5): Cost factor has a negative impact on fertility intentions. Among the demographic characteristics, housing conditions have a moderating effect.
Hypothesis 6 (H6): Utility factor has a positive impact on attitude. Among the demographic characteristics, age, gender, age of the first child, and gender of the first child have moderating effects.
Hypothesis 7 (H7): Utility factor has a positive impact on fertility intentions. Among the demographic characteristics, age, gender, age of the first child, and gender of the first child have moderating effects.
Hypothesis 8 (H8): Parental help has a positive impact on attitude.
Hypothesis 9 (H9): Parental help has a positive impact on perceived behavior control.
Hypothesis 10 (H10): Attitude has a positive impact on fertility intentions.
Hypothesis 11 (H11): Subjective norms have a positive impact on fertility intentions.
Hypothesis 12 (H12): Perceived behavior control has a positive impact on fertility intentions.

**Fig 1:**
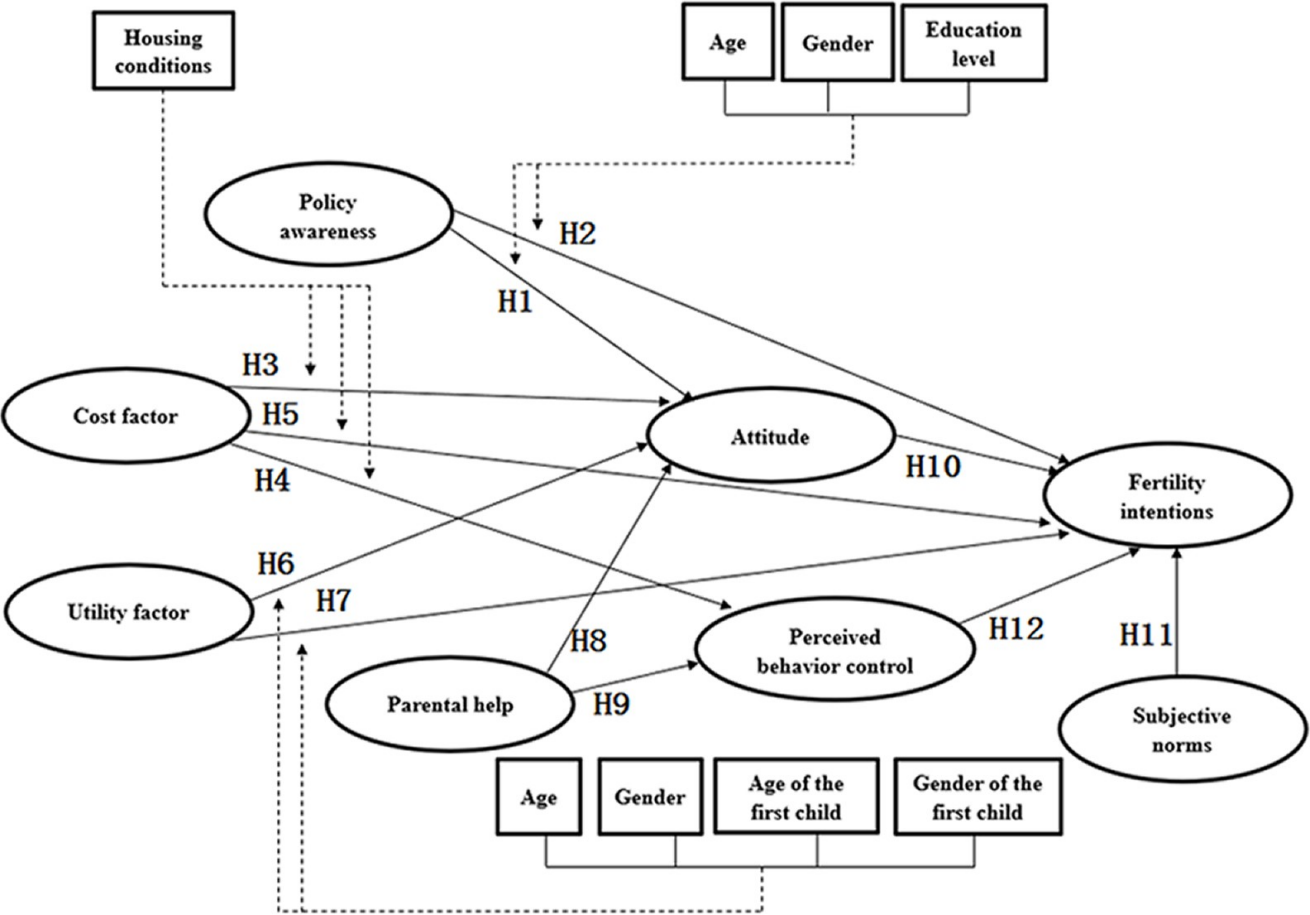
Original model diagram.

The research model in Fig 1 was constructed using the influencing factors and the research hypotheses and contains 7 independent variables, 1 dependent variable and 7 moderating variables, and 12 research hypotheses.

## Methodology

### Questionnaire design

The questionnaire included two main parts. The first collected the participants’ basic demographic information using seven multiple choice questions on gender, age, health status, education level, housing conditions, gender of the first child, and age of the first child. The questions in this part corresponded to the moderating variables in Fig 1. The second part recorded information about the factors influencing second child fertility intentions. It contained 24 questions, with 3 questions each on policy awareness, cost factor, utility factor, attitude, subjective norms, perceived behavior control, parental help, and fertility intentions. All 24 questions used a 5-point Likert scale, with higher numbers indicating progressively stronger agreement. The questions in this part correspond to the independent variables and dependent variable. After designing the questionnaire, we carried out a pilot study, distributing the questionnaire to a small number of researchers at Shandong Management University to review the questionnaire content. Due to the small number of researchers, it was not possible to import the data from the pilot study into SPSS for analysis. We modified and improved the questionnaire content based on feedback from the pilot study. Table 2 presents the final 24 questions related to the influencing factors.

**Table 2:** Questions on influencing factors.

Dependent variables
**Policy awareness**
I know about the two-child policy.
I understand the two-child policy very well.
I’m very aware of the specifics of the two-child policy.
**Cost factor**
The financial cost of having a second child is too high and would put me under financial pressure.
The time cost of having a second child is too high and would have an impact on my job.
Having a second child is too much work and would affect my quality of life.
**Utility factor**
Having a second child may provide financial security for my future retirement.
Having a second child may bring more joy and fulfillment to my family.
Multiple children can take care of each other, avoiding one child being alone.
**Attitude**
I fully support the two-child policy.
I think the two-child policy is a good policy.
I think the two-child policy is very important.
**Subjective norms**
My parents think I should have a second child if it is in line with policy.
My friends think I should have a second child if it is in line with policy.
My coworkers think I should have a second child if it is in line with policy.
**Perceived behavior control**
My financial situation allows me to have a second child.
I have enough time to have a second child.
I have enough energy to have a second child.
**Parental help**
My parents’ financial help could enable me to have a second child.
My parents’ help could give me the time to have a second child.
My parents’ energetic help could enable me to have a second child.
**Fertility intentions**
I will pay more attention to the policies and knowledge about the birth of a second child.
I plan to someday have a second child.
I plan to have a second child within the next few years.

### Data collection

The non-probability sampling method of convenience sampling was used to recruit participants. The advantages of this method are that it is relatively simple and easy to conduct, and the input cost of data collection is relatively low [114]. Based on the study intentions, the participants were all teachers from Jinan who have similar social backgrounds and economic status, so that those participant characteristics would not have an impact on the results. From this point of view, the use of convenience sampling for this study can be justified. Online questionnaires were distributed among faculty at several colleges in Jinan, China, including Shandong Management University, Shandong Women’s University, Shandong Normal University, Qilu University of Technology, University of Jinan, Shandong University of Art & Design, and Shandong College of Arts. Data was collected from February 1, 2021 to February 28, 2021, and a total of 213 valid questionnaires were collected. Since this paper focused on second child fertility intentions, the age range for participants (judged most likely to have a second child) was set between 20 and 50, and responses from participants younger than 20 or older than 50 were discarded, leaving a total of 194 questionnaires for analysis.

### Data analysis

**Fig 2:**
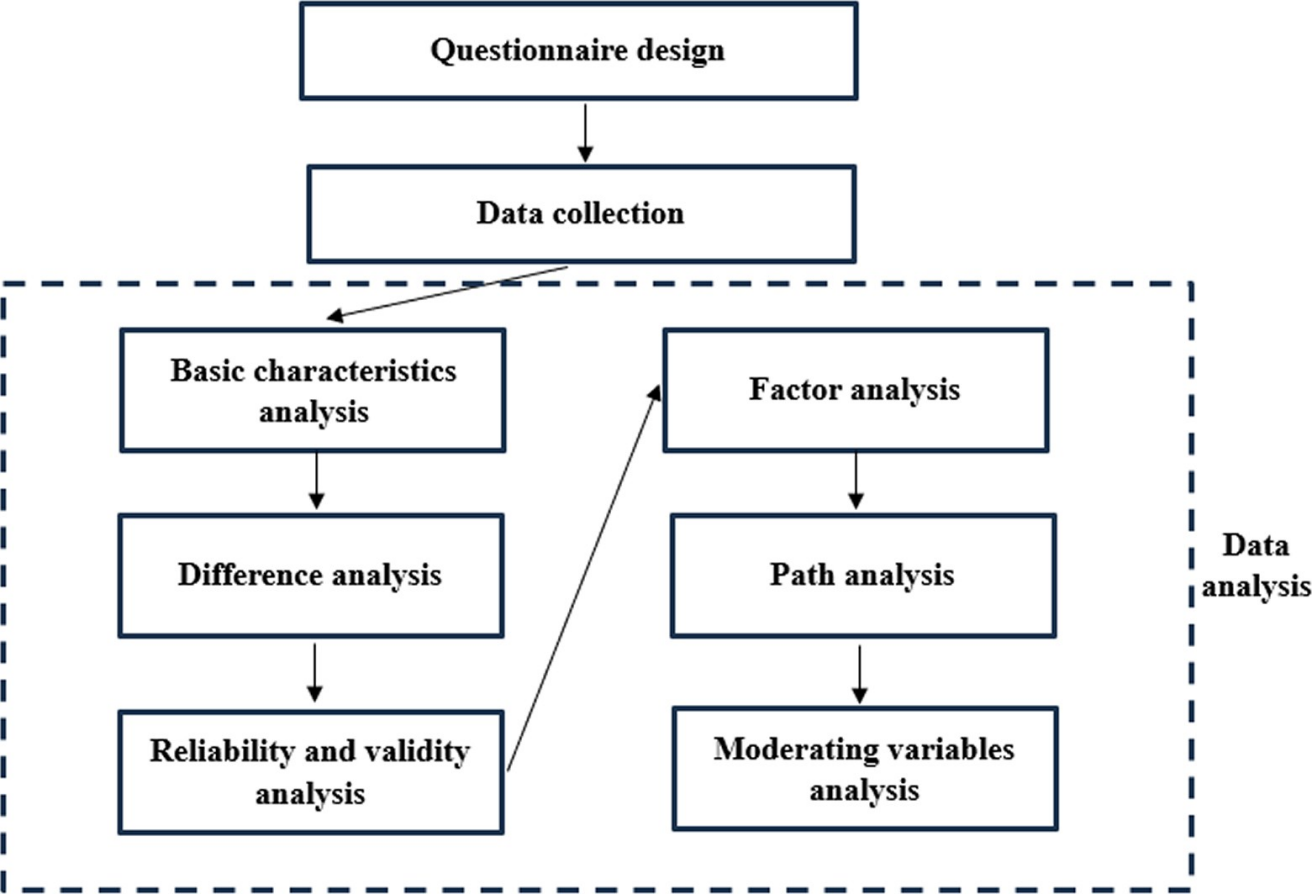
Flow diagram.

The data was imported to SPSS for analysis including basic characteristics analysis, difference analysis, reliability and validity analysis, factor analysis, path analysis and moderating variables analysis. The flow diagram is shown in Fig 2.

### Ethics statement

This study was approved by the Academic Committee of Shandong Management University (approval number XS0001). At the beginning of the questionnaire, a written explanation was provided “If you agree to participate, please truthfully fill in and submit based on your actual situation and first impression. If you do not agree, please ignore this questionnaire and do not fill it in.” Therefore, participants who completed the questionnaire are considered to be consenting. This study did not involve minors. Individual participants could not be identified by the questionnaire data obtained, thus ensuring the privacy of the participants.

## Findings

### Basic characteristics analysis

The results of the basic characteristics analysis are shown in Table 3. Among the participants, 62.9% were women and 37.1% were men. The largest age group among respondents was 31–40 years old, accounting for 61.3%, followed by 41–50 years old, accounting for 34.0%. The proportion of 21–30 years old was the lowest, accounting for 4.6%. Over half of the respondents (54.1%) self-reported poor health; those who reported average health accounted for 42.3%, and those who reported good health constituted only 3.6%. For education level, the highest number of participants (62.9%) had a master’s degree, followed by doctorate (20.6%), bachelor’s (15.5%), and junior college (1.0%). Most participants lived in self-purchased commercial housing (74.7%); other housing types included employer or family provided housing (9.3%), rental housing (7.2%), others (6.2%), office temporary housing (1.5%), government low-rent housing (0.5%), and government public rental housing (0.5%). For gender of the first child, 62.9% were boys and 37.1% were girls. Most first children were primary school age (33.0%), kindergarten age (20.1%), and junior high school age (19.1%), followed by preschool age (1–3 years old; 10.3%), college age (8.8%), infants (under 1 year old; 5.2%), and senior high school age (3.6%).

**Table 3:** Basic characteristics of participants (n = 194).

Variables	n	%
**Gender**		
Men	72	37.1
Women	122	62.9
**Age**		
21–30 years old	9	4.6
31–40 years old	119	61.3
41–50 years old	66	34.0
**Health status**		
Good	7	3.6
Average	82	42.3
Poor	105	54.1
**Education level**		
Junior college	2	1.0
Bachelor’s	30	15.5
Master’s	122	62.9
Doctorate	40	20.6
**Housing conditions**		
Employer or family provided housing	18	9.3
Government low-rent housing	1	0.5
Government public rental housing	1	0.5
Self-purchased commercial housing	145	74.7
Rental housing	14	7.2
Office temporary housing	3	1.5
Others	12	6.2
**Gender of the first child**		
Boy	122	62.9
Girl	72	37.1
**Age of the first child**		
Infant (under 1 year old)	10	5.2
Preschool age (1–3 years old)	20	10.3
Kindergarten age	39	20.1
Primary school age	64	33
Junior high school age	37	19.1
Senior high school age	7	3.6
College age	17	8.8

### Difference analysis

The results of difference analysis are shown in Table 4. There were significant differences in fertility intentions based on age and age of the first child; the fertility intentions of the 31–40 years old participants were significantly higher than those 41–50 years old. Table 5 shows average value of fertility intentions by participants’ age and age of the first child. The fertility intentions of participants whose first child was college age were significantly lower than those of participants whose first child was in a different age group. Table 5 shows the relationship between fertility intentions and age and fertility intentions and age of the first child. Fertility intentions decrease continuously as age increases, and fertility intentions fluctuate as age of the first child increases. The participants whose first child was junior high school age and kindergarten age had the highest second child fertility intentions.

**Table 4:** Analysis results of differences in fertility intentions by participants’ age and age of the first child.

Moderating variable	Difference	Asymptotic significance result	Value	Average value
Age	Difference 1	0.01	31–40 years old	104.51
			41–50 years old	81.89
Age of the first child	Difference 1	0.01	Infant (under 1 year old)	97.85
			College age	50.94
	Difference 2	0.00	Preschool age (1–3 years old)	101.93
			College age	50.94
	Difference 3	0.00	Kindergarten age	113.46
			College age	50.94
	Difference 4	0.01	Primary school age	91.83
			College age	50.94
	Difference 5	0.00	Junior high school age	113.80
			College age	50.94

**Table 5:** Average value of fertility intentions by participants’ age and age of the first child.

Moderating variable	Value	Average value
Age	21–30 years old	119.28
	31–40 years old	104.51
	41–50 years old	81.89
Age of the first child	Infant (under 1 year old)	97.85
	Preschool age (1–3 years old)	101.93
	Kindergarten age	113.46
	Primary school age	91.83
	Junior high school age	113.80
	Senior high school age	74.21
	College age	50.94

### Reliability and validity analysis

The results of the reliability and validity analysis are shown in Table 6; results indicate the data in the second part of the questionnaire was suitable for factor analysis.

**Table 6:** Reliability and validity analysis results.

Measurement	Value
Cronbach’s Alpha	0.84
Kaiser-Meyer-Olkin sampling fitness scale	0.71
Bartlett spherical test significance	0.00

### Factor analysis

The results of factor analysis are shown in Tables 7 and 8. The level of communality of the variables in the factor analysis plays an important role in the sample size; when the level of communality is high (usually greater than or equal to 0.6), the impact produced by small sample size will be very low; when the communality is around 0.5, the sample size should be increased appropriately, between 100 and 200 [115]. As can be seen from Table 7, 31 questions have a communality greater than 0.6 and only one question has a communality of 0.5, indicating that despite the small sample size of this paper, it is still suitable for factor analysis. Results of the common method variance test and the factor analysis test show that six factors with eigenvalues greater than 1 are obtained, and these factors can explain 73.64% of the total variance, with the largest factor explaining 26.20% of the total variance. As this data does not explain more than half of the total variance, there is no common method bias. Results of the rotated component matrix of factor analysis are shown in Table 8 (middle columns). Based on the analysis results, the questions in the second part of the questionnaire were adjusted repeatedly, and the newly included influencing factors are shown in Table 8 (right columns). As can be seen from Table 8, questions 25 and 30 were deleted, and the influencing factors associated with some of the questions were adjusted. Eight influencing factors in the second part of the questionnaire were recombined into six influencing factors. Two influencing factors, utility factor and parental help, were deleted, and six influencing factors (i.e., policy awareness, cost factor, attitude, subjective norms, perceived behavior control, and fertility intentions) were retained.

**Fig 3:**
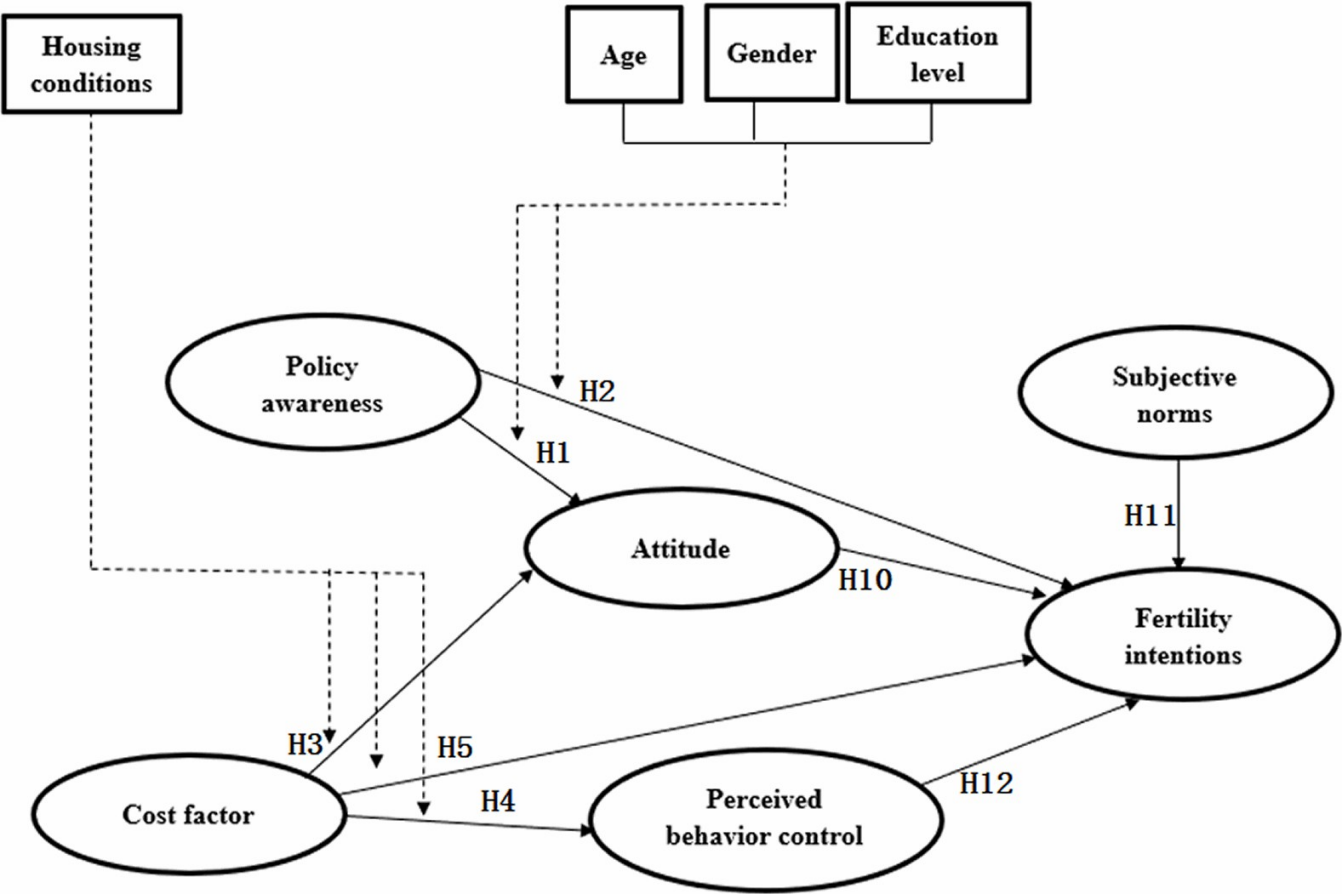
Model diagram after factor analysis.

The original hypotheses and conceptual model were adjusted to account for the removal of the two influencing factors of utility factor and parental help. H6 and H7, related to utility factor, and H8 and H9, related to parental help, were deleted, and the remaining hypotheses retained. The new model is shown in Fig 3.

**Table 7:** Analysis results of communality.

Question #	Initial	Extraction
8	1	0.90
9	1	0.85
10	1	0.78
11	1	0.72
12	1	0.76
13	1	0.73
14	1	0.61
15	1	0.75
16	1	0.71
17	1	0.85
18	1	0.79
19	1	0.84
20	1	0.69
21	1	0.88
22	1	0.63
23	1	0.70
24	1	0.66
25	1	0.67
26	1	0.51
27	1	0.73
28	1	0.78
29	1	0.68
30	1	0.78
31	1	0.69

**Table 8:** Analysis results of rotated component matrix.

Question #	Original influencing factors	Factor analysis rotated component matrix						Processing method	Newly included influencing factors
		1	2	3	4	5	6		
28	Parental help	0.78						Retain	Fertility intentions
29	Fertility intentions	0.70						Retain	
31	Fertility intentions	0.70						Retain	
14	Utility factor	0.68						Retain	
16	Utility factor	0.60						Retain	
26	Parental help	0.52						Retain	
27	Parental help	0.38						Retain	
30	Fertility intentions		0.85					Delete	-
24	Perceived behavior control		0.66					Retain	Perceived behavior control
23	Perceived behavior control		0.45					Retain	
25	Perceived behavior control						-0.37	Delete	-
8	Policy awareness			0.93				Retain	Policy awareness
9	Policy awareness			0.91				Retain	
10	Policy awareness			0.74				Retain	
18	Attitude			0.39				Retain	
17	Attitude				0.91			Retain	Attitude
19	Attitude				0.83			Retain	
15	Utility factor				0.70			Retain	
21	Subjective norms					0.90		Retain	Subjective norms
22	Subjective norms					0.61		Retain	
20	Subjective norms					0.55		Retain	
11	Cost factor						0.82	Retain	Cost factor
13	Cost factor						0.81	Retain	
12	Cost factor						0.49	Retain	

### Path analysis

#### First path analysis

**Fig 4:**
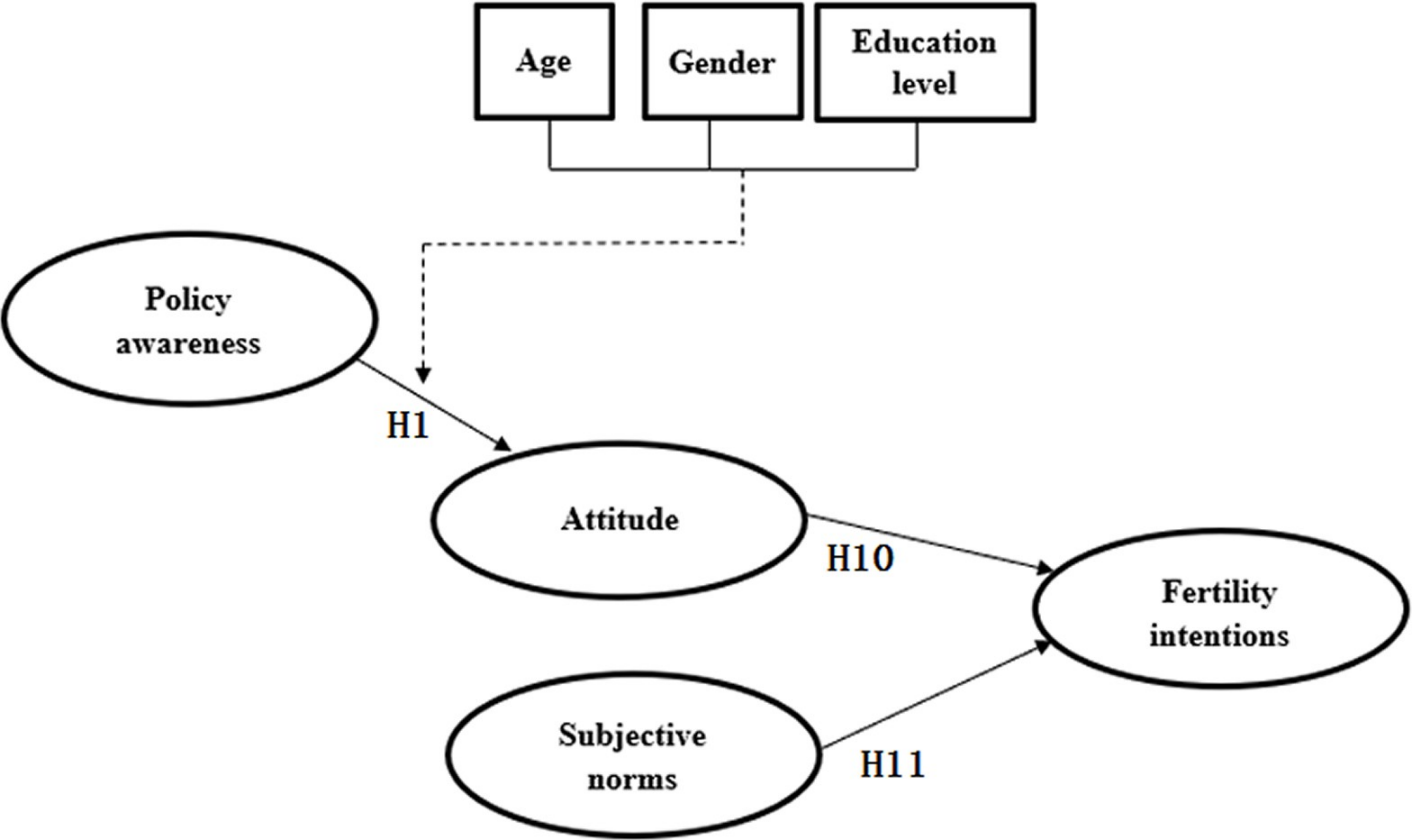
Model diagram after the first path analysis.

The results of the first path analysis are shown in Table 9 (upper section). The analysis of variance (ANOVA) significance of A1, A2, and A3 were all less than 0.05, so they were statistically significant. The p-value of the H1 path in A1, the H4 path in A2, the H10 path, and the H11 path in A3 were all less than 0.05. Therefore, the H1, H4, H10, and H11 paths were retained, while the p-value of other paths was greater than 0.05, so they were deleted (Fig 3). Since the H12 path was deleted, it is of no practical significance to point only to the H4 path of perceived behavior control; the H4 path was deleted, and the related cost factor and perceived behavior control variables were also deleted. The model was further adjusted, deleting H3, H4, and H5, related to cost factor, and H12, related to perceived behavior control. The resulting model is shown in Fig 4.

**Table 9:** Results of the first path analysis and the second path analysis.

Analysis	Step	Dependent variable	R^2^	ANOVA	Independent variable /path	Standardized Beta coefficient	P-value
First path analysis	A1	Attitude	0.24	0.00	Policy awareness /H1	0.49	0.00
					Cost factor /H3	0.01	0.87
	A2	Perceived behavior control	0.07	0.00	Cost factor /H4	-0.26	0.00
	A3	Fertility intentions	0.24	0.00	Policy awareness /H2	-0.04	0.63
					Cost factor /H5	-0.11	0.11
					Attitude /H10	0.18	0.02
					Subjective norms/H11	0.37	0.00
					Perceived behavior control /H12	0.02	0.82
Second path analysis	A4	Attitude	0.24	0.00	Policy awareness /H1	0.49	0.00
	A5	Fertility intentions	0.22	0.00	Attitude /H10	0.16	0.03
					Subjective norms/H11	0.39	0.00

### Second path analysis

The results of second path analysis are shown in Table 9 (the bottom section). The ANOVA significance of A4 and A5 was less than 0.05, so they are statistically significant. From the perspective of p-value, the p-value of the H1 path in A4, the H10 path, and the H11 path in A5 is less than 0.05, so the H1, H10, and H11 paths in Fig 4 were retained. After the second path analysis, the final remaining hypotheses in the conceptual model were H1, H10, and H11.

Based on the standardized Beta coefficient in Table 9, the final influencing coefficients of policy awareness, attitude, and subjective norms on fertility intentions were calculated after the second path analysis. Among them, the influencing coefficient of subjective norms on fertility intentions was the highest, with a value of 0.39, the influencing coefficient of attitude was 0.16, and the influencing coefficient of policy awareness was the lowest, with a value of 0.49 * 0.16 = 0.08.

### Moderating variables analysis

The moderating effect can be expressed as an interaction between an independent variable and a factor that specifies the appropriate conditions under which it operates, and the moderating variable is a third variable that affects the correlation between the other two variables. As can be seen in Fig 4, of the H1, H10, and H11 paths, only the H1 path is affected by the moderating variables of age, gender, and education level. The hierarchical linear regression method was used to analyze the moderating variables, and the results of the moderating variables analysis are shown in Table 10.

**Table 10:** Analysis results of moderating variables.

Model	Variable type	Variables	R^2^	ANOVA	Standardized Beta coefficient	P-value
Model1	Independent variable	Policy awareness	0.26	0.00	0.48	0.00
Model2	Independent variable	Policy awareness	0.33	0.00	3.67	0.00
	Interaction between independent variable and moderating variable	Policy awareness * Age			-2.01	0.00
		Policy awareness * Gender			-0.84	0.06
		Policy awareness * Education level			-1.33	0.01

Model 1 in Table 10 does not include the interaction item of the moderating variable, while Model 2 in Table 10 includes the interaction item of the moderating variable. From Table 10, it can be seen that from Model 1 to Model 2, the R^2^ value increases from 0.26 to 0.33, indicating that the explanatory power of Model 2 is enhanced after the addition of the moderating variable, so the moderating variable had a moderating effect. From the p-value of Table 10, the p-value of policy awareness * age and policy awareness * education level in Model 2 is less than 0.05, indicating that the two moderating variables of age and education level had significant moderating effects, while the p-value of policy awareness * gender is greater than 0.05, indicating that the gender moderating variable was not significant. From the standardized Beta coefficient in Table 10, the absolute values of the standardized Beta coefficients of policy awareness, policy awareness * age, and policy awareness * education level are all greater than 1, and all of them exceed the critical value of the standardized Beta coefficient of -1 to 1, indicating that the two moderating variables of age and education level both have collinearity problems. These results indicate the variables of age, gender, and education level had no moderating effect, so Model 2 was abandoned.

**Fig 5:**
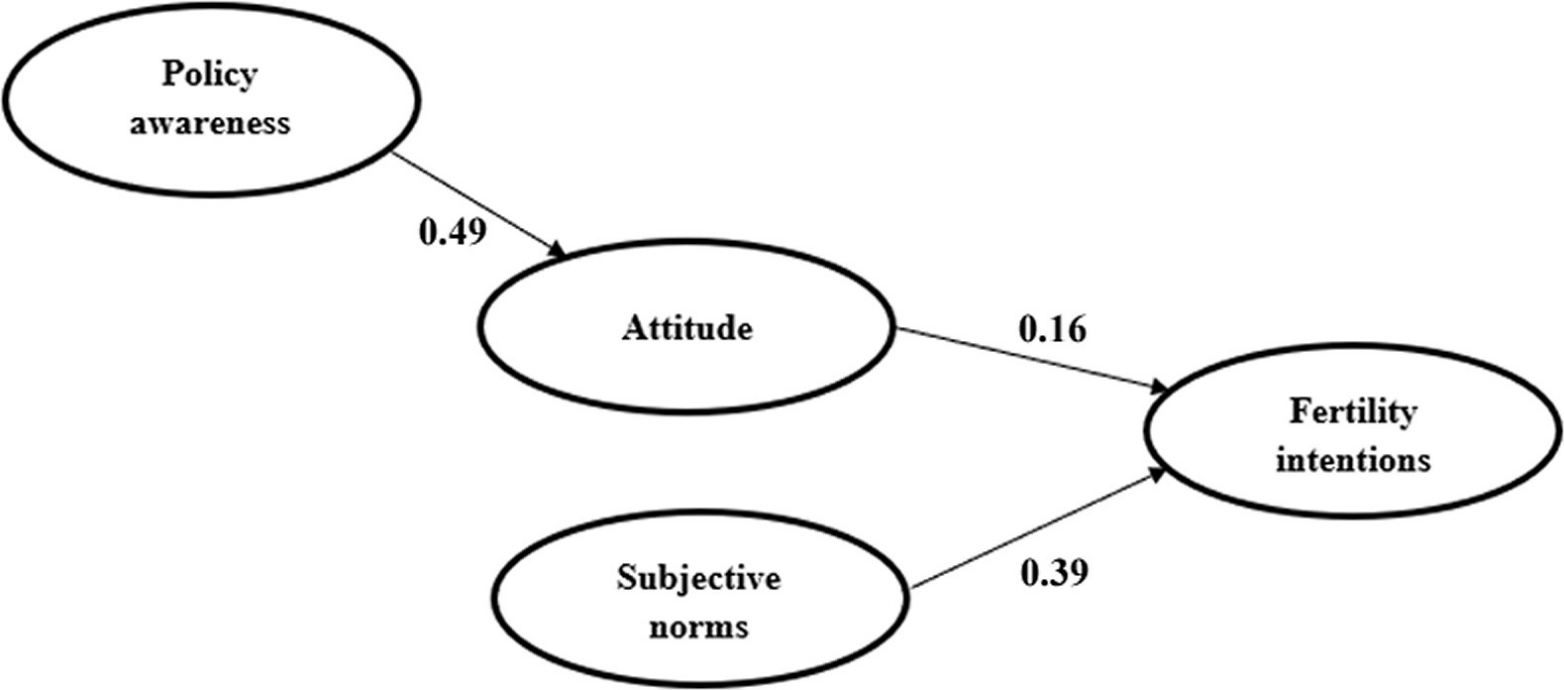
Final model diagram.

The final model combined the results of the path analysis and the moderating variables analysis and is shown in Fig 5. Independent variables include policy awareness, attitude, and subjective norms, and the dependent variable is fertility intentions. Among the three influencing factors, subjective norms had the greatest impact on fertility intentions (0.39), followed by attitude (0.16), and policy awareness had the lowest impact on fertility intentions (0.08).

## Discussion

This paper examines the factors influencing college teachers’ second child fertility intentions in Jinan, China. Specifically, this paper focuses on whether factors suggested by the TPB—attitude, subjective norms, and perceived behavior control—as well as policy awareness, cost factor, utility factor, and parental help, play a role in college teachers’ second child fertility intentions, and how these influencing factors affect each other. In addition, this paper considers the moderating effects of age, gender, education level, housing conditions, age of the first child, and gender of the first child.

This study’s participants were college teachers, most 30–40 years old with master’s or doctorate degrees, with more women than men in the sample. Their self-reported health status was generally poor, which was generally consistent with previous findings of generally poor mental health or physical health in the teacher population [116–118]. Most lived in housing that they owned, and their first child was most commonly a boy, 3–14 years old. Although scholars have suggested that the ideal time for women to have a first child is 23 years old in terms of biological factors [119], studies have found that the peak age for women to have a child is 25–34 years old in terms of biological factors [120], so this study’s participant age distribution is consistent with the age when many women would likely be considering a second child.

Examining differences in fertility intentions by age, participants in the 31–40 years old range had significantly higher second child fertility intentions than participants in 41–50 years old range, as might be expected based on the most common age range for having a second child. There was a difference of about five years from the previous conclusion that the peak age of fertility was between 25 and 34 years old [121], which might be mainly due to the fact that this paper explored the second child fertility intentions, and therefore the peak age of childbearing was shifted by five years. Second child fertility intentions decreased as age increased. Participants whose first child was college age had significantly lower second child fertility intentions than participants whose first child was in another age range.

From the perspective of specific influencing factors, policy awareness had a positive impact on attitude, which indirectly affected second child fertility intentions. Attitude had a positive impact on second child fertility intentions, which is consistent with the conclusions of previous scholars that both attitude and subjective norms have an impact on second child fertility intentions [50,122]. However, in contrast to previous studies, this study did not find that perceived behavior control had a significant impact on second child fertility intentions [50,122]. Combined with the results in basic characteristics analysis, the reason for the lack of a significant effect of perceived behavior control on second child fertility intentions could be due to the relatively poor health status of the participants. Poor health status, and the fact that college teachers generally undertake more scientific research, may lead college teachers to weigh more heavily the energy, physical strength, and time required in the decision of whether to have a second child. Among the three TPB core influencing factors, only perceived behavior control had no significant impact on second child fertility intentions. This indicates TPB offers an appropriate analytical framework for the study of fertility intentions [108], and suggests the application of TPB to understanding second child fertility intentions among college teachers was reasonable. In addition, among the four other factors considered (policy awareness, parental help, cost factor, and utility factor), only policy awareness had a significant effect on second child fertility intentions indirectly, which also suggests that the use of TPB can provide a better research explanation of fertility intentions and deepen the understanding of fertility decisions [109]. This is not consistent with previous findings on the relationship between parental help and second child fertility intentions [32].

From the perspective of the importance of influencing factors, previous studies have shown that the most important factors affecting second child fertility intentions are age and the number of siblings, respectively [33], whereas this paper draws a different conclusion. Subjective norms had the greatest impact on second child fertility intentions, followed by attitude, and policy awareness had the least influence. Previous studies have demonstrated that subjective norms derived from social relations such as relatives, friends, and colleagues have a significant impact on fertility behavior and fertility intentions [122,123], and this paper’s findings provide evidence that subjective norms have an important impact on the second child fertility intentions of college teachers. This finding suggests relatives, friends, and colleagues play an important role in influencing college teachers to have a second child. Previous studies have shown that attitude plays an important role in fertility intentions [108,122], and this paper also suggests attitude plays an important role in second child fertility intentions, but the attitude’s influence is not as strong as that of subjective norms. Previous research indicates that positive and stable fertility policy has positive implications for sustainable population development, beneficial economic and social impacts, and important implications for individual fertility decisions [124]. This paper’s findings also support this view. The key to effective public policy implementation lies in its effective dissemination and efforts to maximize policy awareness [125]. From the findings of this paper, the sample population of college teacher had a high degree of awareness and acceptance of fertility policy. Future research could further explore the effect of fertility policies on fertility intentions in other groups.

### Implications

#### Theoretical implications

The findings of this paper show that subjective norms have an important role in influencing college teachers’ second child fertility intentions, suggesting parents, relatives, friends, and colleagues may influence this decision. Further research should consider the relative influence of each of these groups and both the effects of subjective norms and the mechanisms of their interaction. Moreover, these findings indicate fertility policy influences college teachers’ second child fertility intentions. As college teachers have a strong sensitivity to and a high degree of knowledge about fertility policy, fertility policy may have a greater impact on this population’s decisions than on the general population’s second child fertility intentions. Comparing the effects of fertility policy among various populations offers a key avenue for future research.

#### Practical implications

The results of this study show that fertility policies, subjective norms, and attitude all have an impact on college teachers’ second child fertility intentions. These findings suggest the following practical implications.

First, government departments and education departments should give full consideration to the various childbirth and child-rearing needs of college teachers of childbearing age and create a work environment for college teachers that is more suitable for childbearing. Providing college teachers, especially female college teachers, with a more congenial working environment and space for career development can alleviate the problem of feeling forced to choose between work and childbirth and child-rearing, and effectively improve the overall fertility level of college teachers.

Second, in addition to considering the role of fertility policy in solving the macro-level issues of rapid population aging, low birth rate, and shrinking labor force, policymakers also need to fully consider and address the role of fertility policy in influencing fertility attitude and intentions at the individual level. In formulating fertility policy, policymakers should comprehensively consider the actual fertility needs of college teachers and formulate and implement relevant policies accordingly.

Third, these findings show that college teachers have a high degree of sensitivity to and recognition of fertility policies, and that better knowledge and recognition of fertility policies are associated with more positive fertility intentions. It is important to create a favorable atmosphere for encouraging childbirth among college teachers and to broaden awareness of the fertility policy among college teachers by leveraging various information technologies.

Fourth, female college teachers usually take on more educational and child-rearing tasks. At the family level, the division of roles regarding family education should be optimized, with husbands taking on more family education tasks and female university teachers better able to balance work and family, which will have a positive effect on family education and fertility intentions.

Finally, various supporting measures should be improved. A fertility information service platform should be set up for college teachers and various kinds of physical and mental health counseling services should be provided to alleviate college teachers’ anxieties about childbirth.

## Limitations and directions for future research

This paper also has some limitations. First, in terms of the selection of influencing factors, we adopted the influencing factors suggested by the TPB and considered additional factors such as policy awareness, cost factor, utility factor, and parental help. In fact, the factors affecting second child fertility intentions are very complex. In order to fully understand the influencing factors of college teachers’ second child fertility intentions, we should not only consider the factors adopted in this paper, but also consider some other factors, such as social welfare and social insurance. Second, participants were all college teachers in Jinan, and the results may not be generalizable outside of this region. Future research should consider additional regions to expand generalizability and further explore the effect of fertility policies on fertility intentions in other groups.

## Research conclusion

This paper explored the factors influencing college teachers’ second child fertility intentions in Jinan, China on the basis of TPB. The results showed that participants between 31 and 40 years old had the highest second child fertility intentions. The factors influencing college teachers’ second child fertility intentions include subjective norms, attitude, and policy awareness. From the theoretical point of view, the results of this study enrich the research on fertility intentions. These findings also can provide an optimal practical reference for the formulation and implementation of China’s fertility policy as well as the improvement of the division of family roles in China.

## Supporting information

S1 Table Relevant data underlying the findings described in manuscript. (SAV)

S2 Table Results of the first path analysis. (XLS)

S3 Table Results of the second path analysis. (XLS)

S4 Table Results of the moderating variables analysis. (XLS)

S1 File Questionnaire. (DOCX)
