# Supplementary material for: Research on influencing factors of college teachers’ second child fertility intentions——Taking Jinan as an example
Source: PLoS One. 2024 May 21;19(5):e0299838. doi: 10.1371/journal.pone.0299838 (PMC11108140; doi:10.1371/journal.pone.0299838)
Supplement: S1 File — (DOCX) [file pone.0299838.s005.docx]

Hello, this questionnaire is about the second child birth, designed by the research group of " Research on the college teachers’ intention to have a second child in Jinan under the comprehensive two child policy - from the perspective of the theory of planned behavior " of Shandong Management University. You do not need to sign your name when answering the questionnaire, your privacy will not be disclosed, and there is no standard answer. The purpose of the questionnaire is to collect and study the situation of second child birth. If you agree to participate, please truthfully fill in and submit based on your actual situation and first impression. If you do not agree, please ignore this questionnaire and do not fill it in. Thank you very much for your support.

**Part one: Basic information**

1、Your gender: A. Men B. Women

2、Your age: A. 21-30 years old B. 31-40 years old C. 41-50 years old

3、Your health status: A. Good B. Average C. Poor

4、Your education level: A. Junior college B. Bachelor’s C. Master’s D. Doctorate

5、Your housing conditions: A. Employer or family provided housing B. Government low-rent housing C. Government public rental housing D. Self-purchased commercial housing E. Rental housing F. Office temporary housing G. Others

6、Your gender of the first child: A. Boy B. Girl

7、Your age of the first child: A. Infant (under 1 year old) B. Preschool age (1-3 years old) C. Kindergarten age D. Primary school age E. Junior high school age F. Senior high school age G. College age

**Part two: Influencing factors**

8、I know about the two-child policy.

A. Strongly disagree B. Disagree C. Neutral D. Agree E. Strongly agree

9、I understand the two-child policy very well.

A. Strongly disagree B. Disagree C. Neutral D. Agree E. Strongly agree

10、I'm very aware of the specifics of the two-child policy.

A. Strongly disagree B. Disagree C. Neutral D. Agree E. Strongly agree

11、The financial cost of having a second child is too high and would put me under financial pressure.

A. Strongly disagree B. Disagree C. Neutral D. Agree E. Strongly agree

12、The time cost of having a second child is too high and would have an impact on my job.

A. Strongly disagree B. Disagree C. Neutral D. Agree E. Strongly agree

13、Having a second child is too much work and would affect my quality of life.

A. Strongly disagree B. Disagree C. Neutral D. Agree E. Strongly agree

14、Having a second child may provide financial security for my future retirement.

A. Strongly disagree B. Disagree C. Neutral D. Agree E. Strongly agree

15、Having a second child may bring more joy and fulfillment to my family.

A. Strongly disagree B. Disagree C. Neutral D. Agree E. Strongly agree

16、Multiple children can take care of each other, avoiding one child being alone.

A. Strongly disagree B. Disagree C. Neutral D. Agree E. Strongly agree

17、I fully support the two-child policy.

A. Strongly disagree B. Disagree C. Neutral D. Agree E. Strongly agree

18、I think the two-child policy is a good policy.

A. Strongly disagree B. Disagree C. Neutral D. Agree E. Strongly agree

19、I think the two-child policy is very important.

A. Strongly disagree B. Disagree C. Neutral D. Agree E. Strongly agree

20、My parents think I should have a second child if it is in line with policy.

A. Strongly disagree B. Disagree C. Neutral D. Agree E. Strongly agree

21、My friends think I should have a second child if it is in line with policy.

A. Strongly disagree B. Disagree C. Neutral D. Agree E. Strongly agree

22、My coworkers think I should have a second child if it is in line with policy.

A. Strongly disagree B. Disagree C. Neutral D. Agree E. Strongly agree

23、My financial situation allows me to have a second child.

A. Strongly disagree B. Disagree C. Neutral D. Agree E. Strongly agree

24、I have enough time to have a second child.

A. Strongly disagree B. Disagree C. Neutral D. Agree E. Strongly agree

25、I have enough energy to have a second child.

A. Strongly disagree B. Disagree C. Neutral D. Agree E. Strongly agree

26、My parents' financial help could enable me to have a second child.

A. Strongly disagree B. Disagree C. Neutral D. Agree E. Strongly agree

27、My parents' help could give me the time to have a second child.

A. Strongly disagree B. Disagree C. Neutral D. Agree E. Strongly agree

28、My parents' energetic help could enable me to have a second child.

A. Strongly disagree B. Disagree C. Neutral D. Agree E. Strongly agree

29、I will pay more attention to the policies and knowledge about the birth of a second child.

A. Strongly disagree B. Disagree C. Neutral D. Agree E. Strongly agree

30、I plan to someday have a second child.

A. Strongly disagree B. Disagree C. Neutral D. Agree E. Strongly agree

31、I plan to have a second child within the next few years.

A. Strongly disagree B. Disagree C. Neutral D. Agree E. Strongly agree
